# Food and Sex-Related Impacts on the Pharmacokinetics of a Single-Dose of Ginsenoside Compound K in Healthy Subjects

**DOI:** 10.3389/fphar.2017.00636

**Published:** 2017-09-13

**Authors:** Lulu Chen, Luping Zhou, Yaqin Wang, Guoping Yang, Jie Huang, Zhirong Tan, Yicheng Wang, Gan Zhou, Jianwei Liao, Dongsheng Ouyang

**Affiliations:** ^1^Department of Clinical Pharmacology, Xiangya Hospital Central South University Changsha, China; ^2^Institute of Clinical Pharmacology, Central South University Changsha, China; ^3^Center of Clinical Pharmacology, The Third Xiangya Hospital, Central South University Changsha, China

**Keywords:** clinical trial, food effect, sex effect, ginsenosides compound K, pharmacokinetics, ChiCTR-IPR-15005787, http://www.chictr.org.cn/index.aspx

## Abstract

**Background and Objectives:** Ginsenoside compound K (CK) is a candidate drug for rheumatoid arthritis therapy. This clinical trial was designed to evaluate the effects of food and sex on the pharmacokinetics of CK and its metabolite 20(S)-protopanaxadiol (PPD).

**Methods:** An open-label, single-center, two-period crossover trial was performed in healthy Chinese subjects (*n* = 24; male = 12, female = 12), randomized to either the fasting overnight or the high-fat meal group before a single 200 mg dose of monomer CK was administered. According to the concentration-time data of plasma and urine samples from each subject, the pharmacokinetic parameters of CK and 20(S)-PPD were calculated and statistically analyzed.

**Results:** A two-way ANOVA test combined with mean plots showed no statistically significant interaction between food and sex. High-fat meal accelerated the absorption of CK, with *t*_max_ being shortened from 3.6 to 2.5 h (*p* = 0.015). In contrast, food significantly increased the C_max_, AUC_last_, and AUC_inf_(*p* < 0.001) with the 90% confidence intervals falling outside of the conventional 0.80–1.25. Females had higher exposure levels of CK than males, but the difference was statistically significant only after a high-fat meal. Of note, CK was rarely excreted in urine. Furthermore, the effects of food and sex were also observed on 20(S)-PPD.

**Conclusion:** High-fat food and sex both had an impact on the disposition of CK *in vivo*, but rather than a significant interaction effect. High-fat food accelerated and increased the absorption of CK, while the exposure of CK was higher in females compared to males. The results indicate that food and sex should be two noteworthy factors in future research on CK.

## Introduction

Rheumatoid arthritis (RA) is a chronic autoimmune disorder which affects approximately 1–2% of the world's population, being two to three times more common in women than in men. All RA patients suffer from mild disability, which can worsen in time and seriously damage the motor function (Ali and Vino, 2016; Cheung and McLnnes, 2017). Even more unfortunately, a large proportion of patients cannot tolerate current therapies and/or become insensitive to continuous exposure, which suggests that it is necessary to develop novel drugs to improve the existing treatment options for RA.

**Figure 1:**
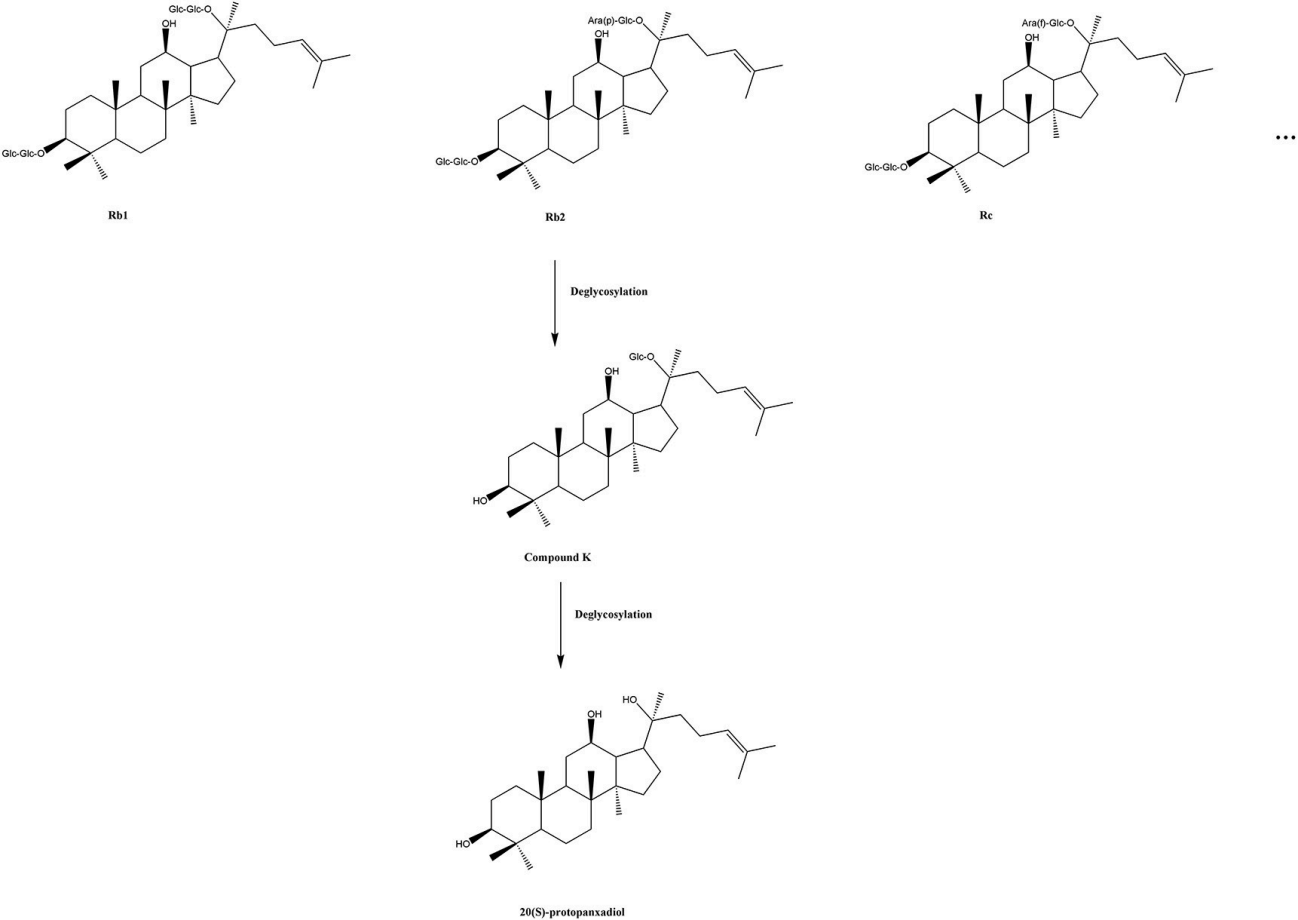
A sketch map of conversion of protopanaxadiol type ginsenosides. Glc, D-glucopyranosyl; Ara(p), L-arabinopyranosyl; Ara(f), D-arabinofuranosyl.

A complex, interactive network of cells and cytokines has been proven to be involved in the pathogenesis of RA (Kurko et al., 2013). A considerable number of pre-clinical studies have shown that ginsenoside compound K (20-O-beta-D-glucopyranosyl-20(S)-protopanaxadiol, also named IH-901, M1 and G-CK) exhibited good anti-inflammatory activity as a glucocorticoid receptor agonist and an inflammatory cytokine inhibitor (Cuong et al., 2009; Joh et al., 2011; Wu et al., 2014). Ginseng is a traditional herbal medicine, extensively used in Asia for its beneficial effects (Yun, 2001), which are primarily attributable to the function of ginsenosides, a group of triterpene saponins (Christensen, 2009). Ginsenoside CK is the main degradation product of protopanaxadiol-type ginsenosides, such as, ginsenoside Rb1, Rb2, Rc, etc., in the human intestine (Akao et al., 1998; Bae et al., 2002; Yang et al., 2015), and it can be further disintegrated by gastric acid and/or intestinal microorganisms into 20(S)-protopanaxadiol (PPD) *in vivo* (Oh and Kim, 2016; Figure 1). Recent studies have found that ginsenoside CK is a compound with multiple targets and pharmacological activities such as, anti-inflammatory (Hossen et al., 2017), anti-tumor (Chen et al., 2016), anti-pruritic (Choo et al., 2003; Shin and Kim, 2005), anti-diabetic (Li et al., 2012), hepatoprotective effect (Wei et al., 2015), etc. Despite this, CK has not been released on the market as a new drug, which could be partially due to its scarcity in nature and difficulties in industrial production. Currently, Ginsenoside CK Tablet is being tested as an anti-RA drug in China, under the approval (CDEL20130379) of the China Food and Drug Administration (FDA) to commence clinical trials (registration numbers: ChiCTR-TTRCC-14004177, ChiCTR-TRC–14004824, ChiCTR-IPR-15005787, and ChiCTR–15006107, on the Chinese Clinical Trial Registry http://www.chictr.org.cn/enindex.aspx). The production of the test drug by Hisun Pharmaceutical Co., Ltd. (Taizhou, China) was based on a method of enzymatic conversion (Yan et al., 2008) combined with an improved macroporous resin purification technology. The content of CK in these tablets is higher than 96.7%.

Research on the pharmacokinetics of CK is particularly limited, with the existing clinical studies (Lee et al., 2009; Jin et al., 2012; Kim, 2013; Kim et al., 2013; Wan et al., 2017) having been performed on ginseng extracts rather than the monomer CK. These studies differ from the present trial, for the generation of CK from extracts *in vivo* involves biotransformation. The only report of monomer CK being tested on humans (Chen et al., 2015) has come from our previous research (registration number: ChiCTR-TTRCC-14004177), and it focused on the liquid chromatography-tandem mass spectrometry (LC-MS/MS) method for the analysis of CK. Furthermore, there are multiple reasons for the necessity to better understand the food-drug interaction(s) before clinical application. Interactions between oral medications and food intake are known to affect drug bioavailability (BA) by reducing, delaying, increasing, or expediting drug absorption (Welling, 1996; Camilleri, 2006). Another important reason is that the disposition of drugs could be influenced by the interactions between nutrients from foods and the transport and/or the drug-metabolizing enzyme systems (Nekvindova and Anzenbacher, 2007; Rodriguez-Fragoso et al., 2011). BA is a key pharmacokinetic (PK) parameter that correlates with the clinical effects of most drugs. On the one hand, unexpectedly increased BA likely results in toxicity, especially for drugs with a narrow therapeutic window. On the other hand, reduced drug BA can lead to ineffectiveness. Warfarin and Alectinib, an anaplastic lymphoma kinase (ALK) inhibitor, are two examples of medication for which food intake must be considered (Hornsby et al., 2008; Morcos et al., 2017). Therefore, the FDA recommends that the food-effect study should be performed, to determine the maximal efficacy with the presence of diet in the gastrointestinal tract for investigational new drugs (INDs). For this purpose, a test meal high in total calories and fat content was recommended (FDA, 2002).

Apart from the effect of diet, our pre-clinical experiments and early clinical trials showed that the pharmacokinetics of CK differed significantly with the sex of the subject (Supplementary Figure S1 and Supplementary Table S1). Therefore, a secondary purpose of this trial is to investigate whether there are any differences in the pharmacokinetics of CK between males and females, and whether the findings are consistent with what was previously observed. This report described an open-label, single-center trial, designed as a two-period crossover study, performed on healthy Chinese subjects (*n* = 24) randomized to either the fasting overnight (FO) or the high-fat (HF) meal group, before receiving a single 200 mg dose of Ginsenoside CK Tablets. Consequently, this study also provides evidence for dosing regimens, for late-stage clinical studies and clinical applications.

## Methods

### Subjects

Subjects were screened for eligibility approximately 1 week before dosing. Screening included medical history, vital signs, physical examination, clinical laboratory tests, and 12-lead electrocardiogram (ECG) recording. Eligibility criteria included being healthy, nonsmoking, non-obese and no-lean (body mass index: 19–24 kg·m^−2^; body weight of females ≥45 kg, males ≥50 kg) male or female subjects, aged 18–45 years. Subjects with any significant, acute, chronic, or infectious disease of the respiratory system, circulatory system, cardiovascular system, digestive system, nervous system, etc. were excluded. Subjects were also excluded for using concomitant treatments (i.e., being within 3 months of exposure to any investigational, over the counter or prescription medications and surgery, and within 30 days of using any drug that inhibits or induces hepatic metabolizing enzymes) or alcohol abuse, etc. In addition, female subjects who were pregnant, nursing, or planning on conceiving, as well as using adequate contraception were not suitable for this trial. All eligible subjects were apprised of the risks of the trial and read, understood, and signed the written informed consent forms prior to participation.

All procedures performed in this trial, involving human participants, were in accordance with the Good Clinical Practice Guidelines, as defined by the International Conference on Harmonization, the Declaration of Helsinki and its later amendments or comparable ethical standards.

### Study designs

The protocol for the food-effect trial was approved by the Institutional Review Board (IRB) of the Third Xiangya Hospital, Central South University, Changsha, China (No.14119).

The food-effect trial was an open-label, randomized, two-period, two-treatment crossover trial, carried out from 15 January to 31 March 2015. Subjects (*n* = 24) were admitted to the clinical facility (Center of Clinical Pharmacology, the Third Xiangya Hospital, Central South University, Changsha, China) and randomized to receive a single 200 mg oral dose of Ginsenoside CK Tablets (4 × 50 mg tablets) either after a 10 h-fasting period or within 30 min after beginning the consumption of a recommended high-fat, high calorie breakfast (150 calories of protein, 250 calories of carbohydrates, 500 to 600 calories of fat; total calories approximately 800–1,000). Tablets were administered with 250 mL of water, to each subject, in each treatment period. Drinking was prohibited for 2 h before and after medication. Apart from this, the amount and time of drinking were not strictly controlled. Subjects had unified, standard meals 4 and 10 h after drug administration. The alternate treatment was conducted after a 14-day washout period.

The dosage of 200 mg of Ginsenoside CK Tablets was chosen as it represents the possible clinical dose. According to the efficacy data of pharmacodynamics studies from our cooperative unit (Chen et al., 2014), CK could exhibit initial anti-inflammatory activity at the dose of 10 mg/kg in rats, which converted to the oral dose in humans is approximately 112 mg. Moreover, results from our previous clinical trial showed that the exposure of CK increased linearly over the dose range of 100–400 mg, with no significant effects on vital signs, laboratory tests or 12-lead ECG (unpublished). Considering that a HF meal may increase CK exposure due to poor solubility, and also for security reasons, a 200-mg dose was selected as the test dose in this trial. Drugs used in this trial were supplied by Hisun Pharmaceutical Co., Ltd. (Taizhou, China) and were preserved under the recommended storage conditions, securely, before being returned to the provider. The composition of the meal was determined based on the FDA guidelines (FDA, 2002) and consisted of one fried egg, two fried fritters, KFC French bacon-egg-pancake and 250 mL of whole milk. The detailed composition and calories of the HF meal used in this trial are shown in Table 1.

**Table 1:** Compositions of the high fat breakfast.

**Nutrient content**	**One KFC french bacon-egg-pancake**	**Two fried fritters**	**One fried egg**	**250 ml whole milk**	**Total (g)**	**Caloric values (Cal)**	**The proportion of total calories (%)**
Protein (g)	14.8	5.6	10	9.7	40.1	160.4	14
Carbohydrates (g)	23.7	45.4	2.5	9.7	81.3	325.2	28
Fat (g)	29.9	18.6	13	11.6	73.1	657.9	58
Total (g)	68.4	69.6	31	25.5	155	1,143.5	100

*1 g protein Calories count by 4 cal; 1 g carbohydrate Calories count by 4 cal; 1 g fat Calories count by 9 cal*.

### Safety assessments

All available information on the study participants was included in the summaries of safety assessments, and must be recorded on the case-report form by investigators. The safety assessment took place throughout the project, and it included continuous observation of adverse events (AEs), monitoring of vital signs, 12-lead ECGs, hematological and biochemical analyses, and urinalysis. Specifically, the monitoring began the night before drug administration (at least 10 h), when subjects were admitted to the clinical facility, and continued for 96 h after dosing. ECGs were recorded prior to the administration and 1, 4, 12, 24, 48, 72, and 96 h after the administration of CK, as well as during physical examinations before and after the trial. Apart from investigators' review, data were also obtained from subjects by recounting or narrating themselves.

Subsequently, AEs were classified by intensity into mild, moderate, and severe. A second classification of AEs, based on the relationship of AEs to the test drug, was performed, dividing them into positive, probable, possible, remote, or unrelated.

### PK assessments

Serial blood samples (5 mL) for PK assessment were collected from an indwelling catheter or by direct venipuncture prior to the administration (*t* = 0) and 0.25, 0.5, 1, 1.5, 2, 2.5, 3, 3.5, 4, 5, 6, 8, 12, 24, 36, 48, 72, and 96 h after the administration of ginsenoside CK. We inferred that these sampling times were sufficient for this trial, based on international guidelines (FDA, 2003; CFDA, 2005) and our previous results, since the sampling time is longer than at least three terminal half-lives of the drug. Additionally, the mean plasma concentrations of both CK and 20(S)-PPD at 96 h were considerably <1/20 of their C_max_ even when the single oral dose at 800 mg (unpublished). Plasma was separated in blood collection tubes by centrifugation (3,000 rpm, 10 min) at 4°C within 1 h after sampling, transferred to labeled storage tubes and stored at −70°C pending workup and analysis.

Urine samples were collected 0–24 and 24–48 h after the administration and the volumes were measured immediately after collection. We preserved 8 mL in centrifuge tube for PK assessment, which were stored at −70°C before analysis.

Descriptive statistics of PK parameters were calculated using established non-compartmental methods, utilizing the WinNonLin version 6.1 software (Pharsight Corporation, USA). The PK parameters determined for each participant included apparent terminal half-life (*t*_1/2_), maximum plasma concentration (C_max_), time to C_max_ (*t*_max_), area under the plasma concentration-time curve from the time of administration up to the last time point with a measurable concentration post-dose (AUC_last_), AUC extrapolated to infinity (AUC_inf_), apparent volume of distribution (V/F), apparent plasma clearance (CL/F) after extravascular administration, mean residence time (MRT_last_ and MRT_inf_), and the cumulative excretion rate from urine. The *t*_1/2_ was estimated as (ln2)/K, where K represented the slope of the terminal phase of plasma concentration-time profile and was assessed with a weighting factor of 1, using least squares (log-linear regression of at least three data points). The C_max_ and *t*_max_ were obtained from experimental observations. The values of AUC were calculated using conventional trapezoidal and log-trapezoidal methods and the extrapolated area (Rowland and Tozer, 1995). CL/F and V/F were calculated as dose/AUC_inf_ and (CL/F)/K, respectively. MRT was calculated as MRT = AUMC/AUC, where AUMC is the area under the first moment curve. The cumulative excretion rate was calculated as the amount of unchanged drug over a time interval in the urine divided by dose, and was expressed as a percentage.

### Analytical methods

All samples were detected using a LC-MS/MS (API 4000, ABI Company, USA) method, validated at the Institute of Clinical Pharmacology, Central South University (Changsha, China). Concentrations of CK and 20(S)-PPD were measured using a triple quadrupole tandem mass spectrometer in multiple reaction monitoring (MRM) mode, via a positive electrospray ionization source at *m/z* 621.4→ 160.8 for the CK and m/z 461.5→425.3 for 20(S)-PPD, with a dwell time of 200 ms.

In brief, after the addition of internal standards (ISs; digoxin for CK, coumarin for PPD), plasma samples (0.5 mL) were deproteinized through a series of procedures, involving the addition of 2 mL of methyl tert-butyl ether (MTBE) and 1 M phosphate buffer, 10 min of mixture and 10 min centrifugation at 4,000 rpm, 4°C. Then 1.4 mL of supernatant were transferred and blow-dried with nitrogen, in the water bath at 40°C. Subsequently, the residue was dissolved in 100 μl of mobile phase and then mechanically shocked, and centrifuged. Finally, the supernatant was kept for injection analysis. CK, 20(S)-PPD, and the ISs were resolved from the matrix components using a HyPURITY C18 (150 × 2.1 mm, 5 μm, Thermo Hypersil-Keystone, USA), with a mobile phase composed of acetonitrile and aqueous ammonium acetate.

The calibration curve range for CK was linear from 1.00 to 1,002.00 ng·ml^−1^ with a correlation coefficient (*r*^2^) equal to 0.9981, and from 0.15 to 54.30 ng·ml^−1^ with *r*^2^ equal to 0.9977 for 20(S)-PPD. Thus, values of the lower limits of quantification (LLOQ) were 1.00 and 0.15 ng·ml^−1^ for CK and PPD, respectively. Accuracy and precision of the assay were determined by evaluating the performance of assay controls. The quality control (QC) samples containing 2.00, 50.10, or 801.60 ng·mL^−1^ of CK and 0.30, 5.43, or 43.44 ng·mL^−1^ of 20(S)-PPD were prepared in human plasma. The accuracy of this LC/MS method was determined by comparing the mean concentrations from each validation run with the theoretical concentrations of components for each QC sample concentrations, while precision was expressed by the coefficient of variation (CV%). According to the results, mean bias values for QCs ranged from −3.15 to 3.56% (CK) and −6.13 to −1.53% (PPD), whereas the CV% values from the QCs for CK and PPD were less than or equal to 9.11%.

The processing of urine samples was similar to that of plasma samples. The calibration curve for CK exhibited excellent linearity from 1.00 to 1,002.00 ng·mL^−1^. For QC samples containing 2.00, 50.10, or 801.60 ng·mL^−1^ CK were prepared using homogenic human urine with the mean bias ranged from −5.54 to 3.56%, while the CVs ranged from 1.38 to 4.89%.

All validation results showed that the method could be used to research the pharmacokinetics of ginsenoside CK in humans.

### Statistical methods

The concentrations of the compounds were measured as mean concentration of each time point and standard deviation (SD), except for *t*_max_, which was expressed as median (range). Major PK parameters such as, *t*_max_, *t*_1/2_, AUC, C_max_, CL/F, V/F, and MRT under fasting or fed conditions were statistically analyzed by SPSS 22.0 (SPSS Inc., USA).

First, a two-way ANOVA combined with interaction plots was conducted to examine the interaction effect of food and sex on the pharmacokinetics of CK. Next, we conducted a *t*-test or Kruskal Wallis test for the PK parameters which showed no interaction effects, to investigate the main effect of sex and food individually. Ninety-percent confidence intervals (CI) for the ratios of geometric means of the exposure parameters were obtained by back transformation, and value *p* < 0.05 was considered significant for any hypothesis tests performed.

## Results

In total, 24 healthy subjects (12 men and 12 women) were enrolled into this trial and randomized between two groups. All subjects received both the HF and FO treatments and completed the trial. Baseline demographics (sex, age, height, weight, and body mass index) across both groups are summarized in Table 2, presented as range and mean ± SD.

**Table 2:** Demographic characteristics of study subjects.

	**Age (year)**	**Height (m)**	**Weight (kg)**	**BMI (kg·m^−2^)**
Male subjects (*n* = 12)	18–29 (23 ± 4)	1.63–1.78 (1.71 ± 0.04)	55.0–76.0 (62.2 ± 6.5)	19.0–24.0 (21.4 ± 1.9)
Female subjects (*n* = 12)	20–28 (23 ± 2)	1.55–1.66 (1.60 ± 0.03)	49.0–64.0 (54.5 ± 4.9)	19.1–23.2 (21.3 ± 1.4)
All subjects	18–29 (23 ± 3)	1.55–1.78 (1.65 ± 0.07)	49.0–76.0 (58.3 ± 6.9)	19.0–24.0 (21.4 ± 1.6)

*BMI, body mass index*.

There were 19 instances of AEs recorded in 11 subjects (45.8%; Table 3). All the AEs were considered mild, and no subject withdrew from the trial. The incidence of AEs classified as positive, probable and possible, in relation to the test drug, was higher in the FO (55.6%) compared to the HF regimen (44.4%). The most commonly reported and drug-related AEs were watery stool (16.7%) and bellyache (12.5%). Furthermore, ginsenoside CK was not associated with any clinically significant abnormalities in physical examination, vital signs, ECGs, or standard clinical laboratory test results.

**Table 3:** Summary of adverse events.

**Adverse events**	**Number of cases**	**Percentage**
Watery stool	4^a^	16.7
Bellyache	3^a^	12.5
Elevated creatine kinase	3^c^	12.5
Elevated triglycerides	3^c^	12.5
Upper respiratory tract infection	1^c^	4.2
Hematuria	1^c^	4.2
Abnormal urine routine	1^c^	4.2
Elevated direct bilirubin	1^b^	4.2
Nausea	1^b^	4.2
Diarrhea	1^b^	4.2

a *Positive related to ginsenoside compound K*.

b *Probable related to ginsenoside compound K*.

c *Possible related to ginsenoside compound K*.

### Interaction effects of food and sex

**Figure 2:**
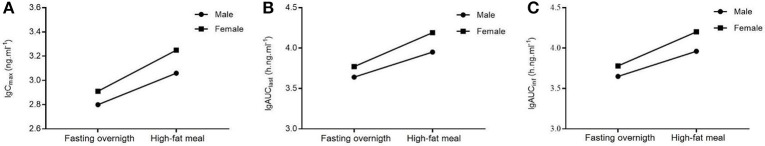
Interaction plots for C_max_ **(A)**, AUC_last_ **(B)**, and AUC_inf_ **(C)** vs. food and sex.

There was no significant interaction effect between food and sex on the main parameters (logarithmic transformed) which measured the exposure levels of CK and 20(S)-PPD (Table 4, analysis results for PPD are not shown). No significant interaction was obtained, with the analysis results [C_max_,*F*_(1, 44)_ = 0.515, *p* = 0.477; AUC_last_, *F*_(1, 44)_ = 0.962, *p* = 0.332; AUC_inf_, *F*_(1, 44)_ = 1.049, *p* = 0.311] based on a two-way ANOVA test. Additionally, interaction plots were used to show how the relationship between diet and PK parameters depends on the sex factor. The mean plots which shows the means for each combination of food and sex for logarithmic transformed C_max_, AUC, V/F, and Cl/F of both CK and 20(S)-PPD were conducted, and no intersection was observed between the two lines in each plot. We chose three graphs as the representative (Figure 2), which clearly shows a similar trend between males and females in the way that food affects the exposure of CK, since the lines are not crossed. Both C_max_ and AUC were highter after the HF meal than under the FO state either in male or female subjects. While female subjects always experienced more CK exposure than males.

**Table 4:** Analysis results from two-way ANOVA to examine the interaction effects on ginsenoside CK between food and sex.

**Parameter**	**Source**	***df***	**Mean square**	***F***	**Sig**.
**CK**					
lgC_max_	Food	1	1.111	26.347	0.000
	Sex	1	0.283	6.699	0.013
	Food ^*^ Sex	1	0.022	0.515	0.477
	Error	44	0.042	na	na
lgAUC_last_	Food	1	1.594	49.384	0.000
	Sex	1	0.394	12.214	0.001
	Food ^*^ Sex	1	0.031	0.962	0.332
	Error	44	0.032	na	na
lgAUC_inf_	Food	1	1.568	50.374	0.000
	Sex	1	0.391	12.55	0.001
	Food ^*^ Sex	1	0.033	1.049	0.311
	Error	44	0.031	na	na
**20(S)-PPD**					
lgC_max_	Food	1	0.36	1.967	0.168
	Sex	1	0.475	2.6	0.114
	Food ^*^ Sex	1	0.183	1.004	0.322
	Error	44	0.183	na	na
lgAUC_last_	Food	1	0.264	1.324	0.256
	Sex	1	1.521	7.637	0.008
	Food ^*^ Sex	1	0.158	0.796	0.377
	Error	44	0.199	na	na
	Total	48			

*CK, compound K; PPD, protopanaxdiol; SD, standard deviation; lg, logarithmic; df, degree of freedom; na, not applicable*.

### Effects of food

All available PK data recorded for each subject were included in the PK assessments, summary statistics and statistical analyses, as predefined in the study protocol.

**Figure 3:**
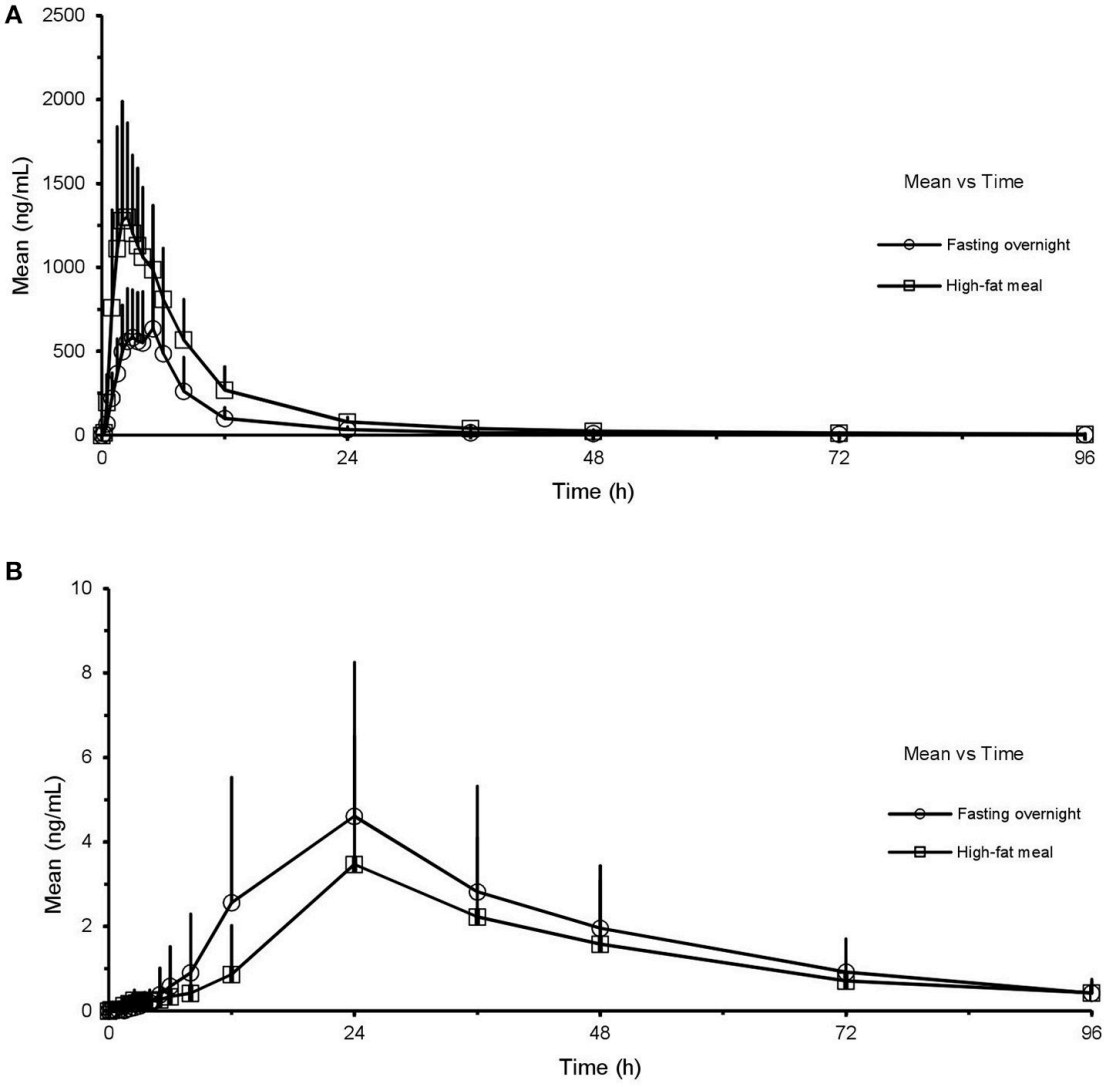
Mean (SD) plasma concentration-time profile of CK **(A)** and 20(S)-PPD **(B)** after oral administration of CK 200 mg in healthy volunteers (*n* = 24) under fasting overnight and after a high-fat meal, respectively. CK, compound K; PPD, protopanaxdiol; All values are presented as mean ± standard deviation.

Mean plasma concentration-time curves of ginsenoside CK and 20(S)-PPD are presented in Figures 3A,B, for the two treatment phases. A summary PK parameters of ginsenoside CK and its metabolite is shown in Table 5, and the summary statistics for the main parameters of CK is tabulated as Table 6.

**Table 5:** Summary pharmacokinetic parameters of ginsenoside CK and 20(S)-PPD.

	**FO (*n* = 24)**	**HF (*n* = 24)**	**FO**		**HF**	
			**Male (*n* = 12)**	**Female (*n* = 12)**	**Male (*n* = 12)**	**Female (*n* = 12)**
**CK**						
*t*_1/2_ (h)	27.7 ± 7.9	24.8 ± 3.0	28.4 ± 10.0	26.9 ± 5.4	24.0 ± 2.4	25.6 ± 3.5
*t*_max_ (h)	3.6 (2.0–6.0)	2.5 (1.5–5.0)	3.4(2.0–5.0)	3.8 (2.0–6.0)	1.5 (1.5–2.5)	2.5 (1.5–4.0)
C_max_ (ng·ml^−1^)	796.8 ± 406.0	1,570.3 ± 587.3	672.2 ± 235.8	921.4 ± 505.1	1,302.0 ± 632.9	1,838.6 ± 404.4
AUC_last_ (h·ng·ml^−1^)	5,748.7 ± 2,830.2	12,599.2 ± 4,098.3	4,749.9 ± 1,718.1	6,747.5 ± 3,408.7	9,633.1 ± 3,313.5	15,565.3 ± 2,223.7
AUC_inf_ (h·ng·ml^−1^)	5,879.3 ± 2,871.0	12,836.7 ± 4,166.2	4,857.1 ± 1,723.2	6,901.4 ± 3,462.0	9,807.0 ± 3,371.8	15,866.5 ± 2,212.7
V/F (L)	1,875 ± 1,899	652 ± 381	2,323 ± 2,572	1,427 ± 699	828 ± 475	477 ± 109
CL/F (L·h^−1^)	43.4 ± 24.2	18.2 ± 9.8	48.8 ± 26.3	38.0 ± 21.8	23.6 ± 11.6	12.8 ± 1.8
MRT_last_ (h)	11.7 ± 1.2	12.3 ± 1.2	11.9 ± 1.3	11.6 ± 1.0	12.4 ± 1.4	12.2 ± 1.0
MRT_inf_ (h)	15.1 ± 4.3	14.6 ± 1.7	15.7 ± 5.7	14.5 ± 2.1	14.5 ± 1.9	14.6 ± 1.5
Cumulative excretion rate (%)	0.060 ± 0.046	0.020 ± 0.015	0.037 ± 0.029	0.083 ± 0.049	0.022 ± 0.020	0.019 ± 0.070
**20(S)-PPD**						
*t*_1/2_ (h)	19.3 ± 6.0^a^	21.2 ± 9.8^a^	18.2 ± 7.1^b^	19.9 ± 5.5^c^	21.2 ± 13.7^b^	21.2 ± 7.6^c^
*t*_max_ (h)	24.5 (12.0–48.0)	26.0 (24.0–36.0)	23.0 (12.0–36.0)	26.0 (12.0–48.0)	26.0 (24.0–36.0)	26.0 (24.0–36.0)
C_max_ (ng·ml^−1^)	5.7 ± 3.8	3.7 ± 3.0	4.9 ± 4.7	6.4 ± 2.7	3.9 ± 3.8	3.5 ± 2.1
AUC_last_ (h·ng·ml^−1^)	161.2 ± 113.7	115.9 ± 97.1	119.6 ± 124.0	202.9 ± 88.8	100.2 ± 98.5	131.5 ± 97.4
AUC_inf_ (h·ng·ml^−1^)	228.1 ± 96.1^a^	162.7 ± 95.4^a^	233.8 ± 120.6^b^	225.0 ± 86.4^c^	178.5 ± 94.2^b^	154.1 ± 99.5^c^
V/F (L)	30,348 ± 22,300^a^	61,539 ± 64,303^a^	25,304 ± 9,268^b^	33,100 ± 27,003^c^	55,230 ± 69,586^b^	64,980 ± 64,481^c^
CL/F (L·h^−1^)	1,067.1 ± 561.9^a^	1,740.1 ± 1,187.7^a^	1,060.5 ± 533.3^b^	1,070.8 ± 602.4^c^	1,417.9 ± 788.7^b^	1,915.9 ± 1,360.0^c^
MRT_last_ (h)	31.7 ± 8.4	33.8 ± 6.9	27.0 ± 7.4	36.3 ± 6.8	30.2 ± 5.9	37.5 ± 5.8
MRT_inf_ (h)	38.5 ± 9.7^a^	45.1 ± 9.5^a^	34.5 ± 12.5^b^	40.7 ± 7.5^c^	42.2 ± 11.1^b^	46.7 ± 8.6^c^

*All values are presented as mean ± SD, except for t_max_, which was expressed as median (range)*.

CK, compound K; PPD, protopanaxdiol; SD, standard deviation; FO, fasting overnight group; HF, high-fat meal group; GMR, test to reference geometric mean ratios; 95% CI, 95% confidence intervals; t_1/2_, terminal half-life; C_max_, maximum plasma concentration; t_max_, time to maximum plasma concentration; AUC_last_, area under the plasma concentration-time curve from zero to the time of the last quantifiable concentration; AUC_inf_, area under plasma concentration-time curve from zero to infinity; V/F, apparent volume of distribution after extravascular administration; MRT_last_, mean residence time from time zero to the time for the last measurable concentration_;_MRT_inf_, mean residence time from time zero to infinity;

a , n = 17;

b , n = 6;

c , n = 11 (accurate determination of t_1/2_, AUC_inf_, V/F, CL/F and MRT_inf_ of PPD were not possible in all subjects because there were less than three data points in the terminal phase so that the parameter K could't be calculated in some subjects)

**Table 6:** Summary statistics for the main pharmacokinetic parameters of ginsenoside CK.

**Parameter**	**Statistical results**	**FO vs. HF *n* = 24**	**FO vs. HF (Male) *n* = 12**	**FO vs. HF (Female) *n* = 12**	**M vs. F (FO) *n* = 12**	**M vs. F (HF) *n* = 12**
**CK**						
*t*_1/2_	*p*-value	0.097	0.158	0.410	0.969	0.308
	GMR (90%CI)	–	–	–	–	–
*t*_max_	*p*-value	0.015	0.123	0.032	0.752	0.937
	GMR (90%CI)	–	–	–	–	–
lgC_max_	*p*-value	<0.001	<0.001	0.001	0.154	0.029
	GMR (90%CI)	0.50 (0.42–0.59)	0.55 (0.46–0.65)	0.45 (0.33–0.62)	0.77 (0.57–1.04)	0.64 (0.46–0.88)
lgAUC_last_	*p*-value	<0.001	<0.001	<0.001	0.116	0.002
	GMR (90%CI)	0.43 (0.36–0.51)	0.49 (0.42–0.57)	0.38 (0.28–0.53)	0.74 (0.54–1.02)	0.59 (0.46–0.75)
AUC_inf_	*p*-value	<0.001	<0.001	<0.001	0.119	0.002
	GMR (90%CI)	0.43 (0.37–0.52)	0.49 (0.42–0.57)	0.39 (0.28–0.54)	0.74 (0.54–1.02)	0.59 (0.46–0.74)
LgV/F	*p*-value	<0.001	<0.001	<0.001	0.166	0.017
	GMR (90%CI)	–	–	–	–	–
LgCL/F	*p*-value	<0.001	<0.001	<0.001	0.119	0.002
	GMR (90%CI)	–	–	–	–	–
MRT_last_	*p*-value	0.059	0.146	0.182	0.814	0.959
	GMR (90%CI)	–	–	–	–	–
MRT_inf_	*p*-value	0.797	0.410	0.906	0.814	0.533
	GMR (90%CI)	–	–	–	–	–
Cumulative excretion rate	*p*-value	<0.001	0.146	0.002	0.015	0.937
	GMR (90%CI)	–	–	–	–	–
**20(S)-PPD**						
lgC_max_	*p*-value	0.015	0.632	0.002	0.097	0.698
	GMR (90%CI)	1.49 (1.15–1.93)	1.12 (0.74–1.70)	1.98 (1.47–2.66)	0.48 (0.23–0.99)	0.84 (0.38–1.84)
lgAUC_last_	*p*-value	0.054	0.767	0.013	0.033	0.290
	GMR (90%CI)	1.41 (1.05–1.88)	1.08 (0.69–1.70)	1.83 (1.27–2.64)	0.34 (0.15–0.75)	0.57 (0.23–1.41)

*CK, compound K; PPD, protopanaxdiol; SD, standard deviation; FO, fasting overnight group; HF, high-fat meal group; GMR, test to reference geometric mean ratios; 90% CI, 90% confidence intervals; lg, logarithmic; t_1/2_, terminal half-life; C_max_, maximum plasma concentration; t_max_, time to maximum plasma concentration; AUC_last_, area under the plasma concentration-time curve from zero to the time of the last quantifiable concentration; AUC_inf_, area under plasma concentration-time curve from zero to infinity; V/F, apparent volume of distribution after extravascular administration; MRT_last_, mean residence time from time zero to the time for the last measurable concentration; MRT_inf_, mean residence time from time zero to infinity; “-”, not determined*.

Except for the *t*_1/2_ and MRT of CK, there were significant differences in the pharmacokinetics of CK between the FO condition and after a HF meal. In the absence of sex factor, *t*_max_ of 24 subjects was shorten from 3.6(2.0–6.0) h to 2.5(1.5–5.0) h with *p* = 0.015 after a HF meal. The C_max_, AUC_last_, and AUC_inf_ of CK all increased about 2-fold compared to the FO condition (C_max_: 1,570.3 ± 587.3 vs. 796.8 ± 406.0 ng·ml^−1^, *p* < 0.001; AUC_last_: 12,599.2 ± 4,098.3 vs. 5,748.7 ± 2,830.2 h·ng·ml^−1^, *p* < 0.001; AUC_inf_: 12,836.7 ± 4,166.2 vs. 5,879.3 ± 2,871.0 h·ng·ml^−1^, *p* < 0.001), with the 90% CIs falling outside of the conventional 0.80 to 1.25. Whereas, V/F and CL/F were decreased in the HF compared to FO subjects (V/F: 652 ± 381 vs. 1,875 ± 1,899 L, *p* < 0.001; CL/F: 18.2 ± 9.8 vs. 43.4 ± 24.2 L·h^−1^, *p* < 0.001). Interestingly, it was observed that ginsenoside CK was rarely excreted in urine. The cumulative excretion rate, which was calculated based on urine concentration-time data, was 0.060 ± 0.046 vs. 0.020 ± 0.015% (*p* < 0.001) in the FO group compared to the HF group. We also investigated the effects of food in male and female subjects, respectively (the last two columns of Table 6). Food exhibited analogous effects on CK in both male and female subjects by significantly increased C_max_, AUC_last_, and AUC_inf_(C_max_: 672.2 ± 235.8 vs. 1,302.0 ± 632.9 ng·mL^−1^ in males, *p* < 0.001; 921.4 ± 505.1 vs. 1,838.6 ± 404.4 ng·mL^−1^ in females, *p* = 0.001; AUC_last_: 4,749.9 ± 1,718.1 vs. 9,633.1 ± 3,313.5 h·ng·mL^−1^ in males, *p* < 0.001; 6,747.5 ± 3,408.7 vs. 15,565.3 ± 2,223.7 h·ng·mL^−1^ in females, *p* < 0.001; AUC_inf_: 4,857.1 ± 1,723.2 vs. 9,807.0 ± 3,371.8 h·ng·mL^−1^ in males, *p* < 0.001; 6,901.4 ± 3,462.0 vs. 15,866.5 ± 2,212.7 h·ng·mL^−1^ in females, *p* < 0.001). Whereas, *t*_max_, CL/F, and V/F were significantly decreased after the HF meal [*t*_max_: 3.4(2.0–5.0) h vs. 1.5(1.5–2.5) h in males, *p* = 0.123; 3.8(2.0–6.0) h vs. 2.5(1.5–4.0) h in females, *p* = 0.032; V/F: 2,323 ± 2,572 vs. 828 ± 475 L in males, *p* < 0.001; 1,427 ± 699 vs. 477 ± 109 L in females, *p* < 0.001; CL/F: 48.8 ± 26.3 vs. 23.6 ± 11.6 L·h^−1^ in males, *p* < 0.001; 38.0 ± 21.8 vs. 12.8 ± 1.8 L·h^−1^ in females, *p* < 0.001]. In addition, the mean *t*_1/2_ was generally comparable for all treatments and ranged from 24.0 to 28.4 h.

As for 20(S)-PPD, it's clear to seen that its exposure was much lower than the parent drug according to the results. According to the Table 5, AUC_last_ was decreased from 161.2 ± 113.7 to 115.9 ± 97.1 h·ng·ml^−1^ in the FO group compared to the HF group, with the GMR (90%CI) was 1.49 (1.15–1.93). The pharmacokinetics of 20(S)-PPD in male subjects did not demonstrate any meaningful difference in either state, while C_max_ and AUC_last_ in females showed significant distinctions (C_max_: 6.4 ± 2.7 vs. 3.5 ± 2.1 ng·mL^−1^, *p* = 0.002; AUC_last_: 202.9 ± 88.8 vs. 131.5 ± 97.4 h·ng·mL^−1^, *p* = 0.013). However, accurate determinations of *t*_1/2_, AUC_inf_, V/F, CL/F, and MRT_inf_ of PPD were not possible in all volunteers (Table 5), because there were less than three data points in the terminal phase so that the parameter K couldn't be calculated in some subjects. The plasma concentrations of PPD that were below the LLOQ at the sampling time after *t*_max_ led to this happening.

### Effects of sex

**Figure 4:**
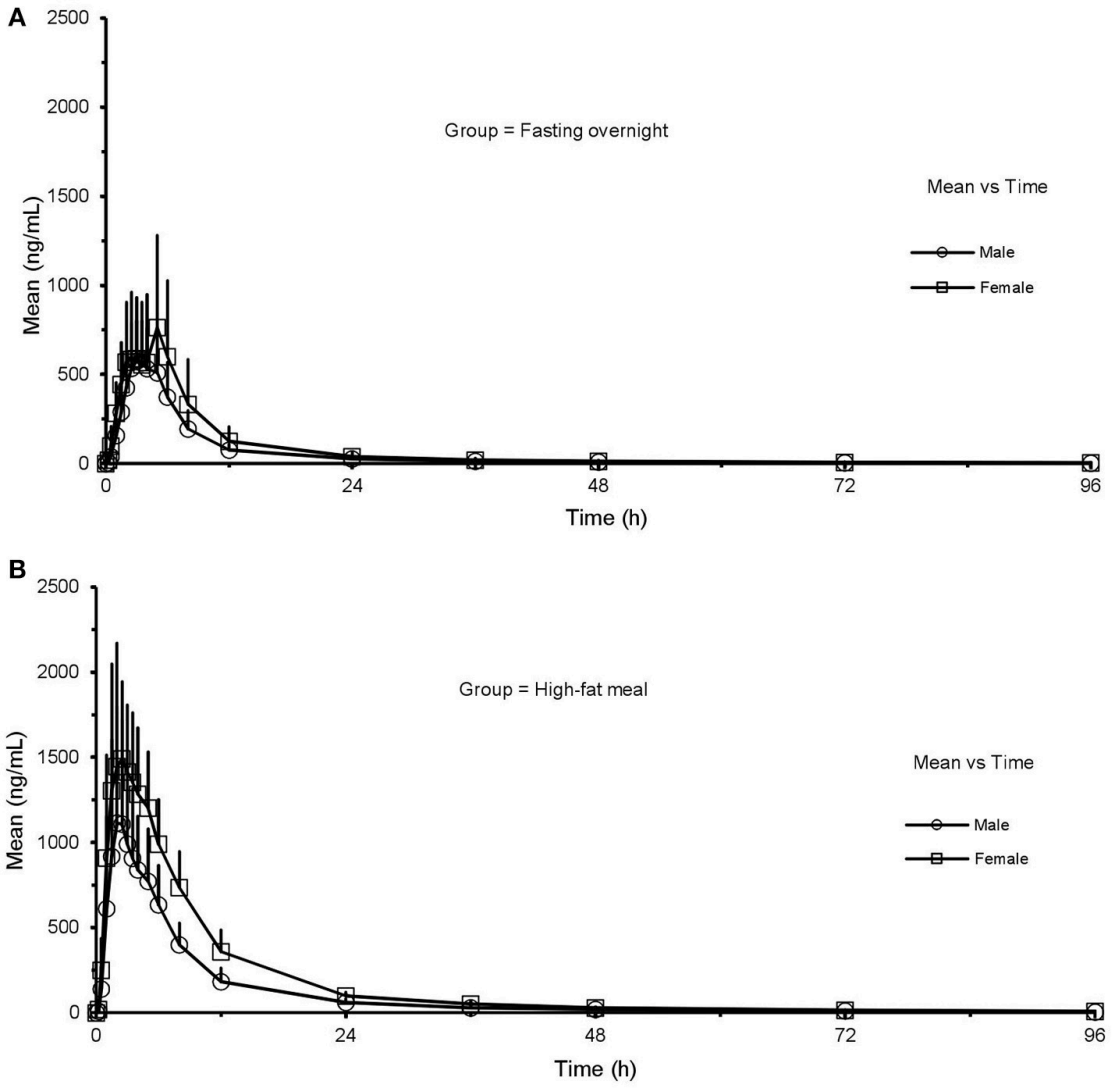
Mean (SD) plasma concentration-time profile of CK after oral administration of CK 200 mg under fasting overnight **(A)** and after a high fat meal **(B)** in male (*n* = 12) and female (*n* = 12) subjects, respectively. CK, compound K; All values are presented as mean ± standard deviation.

Under the FO condition, the C_max_, AUC_last_, and AUC_inf_ of CK were higher in females than in males, with no statistically significant distinctions (C_max_: 921.37 ± 505.14 vs. 672.18 ± 235.76 ng·mL^−1^, *p* = 0.154; AUC_last_: 6,747.48 ± 3,408.66 vs. 4,749.86 ± 1,718.14 h·ng·mL^−1^, *p* = 0.116; AUC_inf_: 6,901.40 ± 3,461.99 vs. 4,857.09 ± 1,723.18 h·ng·mL^−1^, *p* = 0.119, respectively). But the pharmacokinetics of CK was significantly different after a HF meal (Figure 4). Ginsenoside CK was rapidly absorbed after a meal, with 83% (20 of 24) of subjects having measurable CK concentrations at 0.25 h after administration. Table 5 shows the PK parameters of CK in males and females after two different treatments. In the HF group, C_max_, AUC_last_, and AUC_inf_ were significantly higher in females than in males (C_max_: 1,838.61 ± 404.35 vs. 1,301.97 ± 632.93 ng·mL^−1^, *p* = 0.029; AUC_last_: 15,565.27 ± 2,223.72 vs. 9,633.06 ± 3,313.48 h·ng·mL^−1^, *p* = 0.002; AUC_inf_: 15,866.46 ± 2,212.66 vs. 9,807.00 ± 3,371.77 h·ng·mL^−1^, *p* = 0.002), with the ranges of 90% CI confirmed the above distinctions. Moreover, V/F and CL/F were higher in males (V/F: 828 ± 475 L vs. 477 ± 109 L, *p* = 0.017; CL/F: 23.6 ± 11.6 L·h^−1^ vs. 12.8 ± 1.8 L·h^−1^, *p* = 0.002). Contrary to plasma concentration, the sex-related differences in urine appeared in FO state, as 0.083 ± 0.049% in females compared to 0.037 ± 0.029% in males with the value of *p* was 0.015.

**Figure 5:**
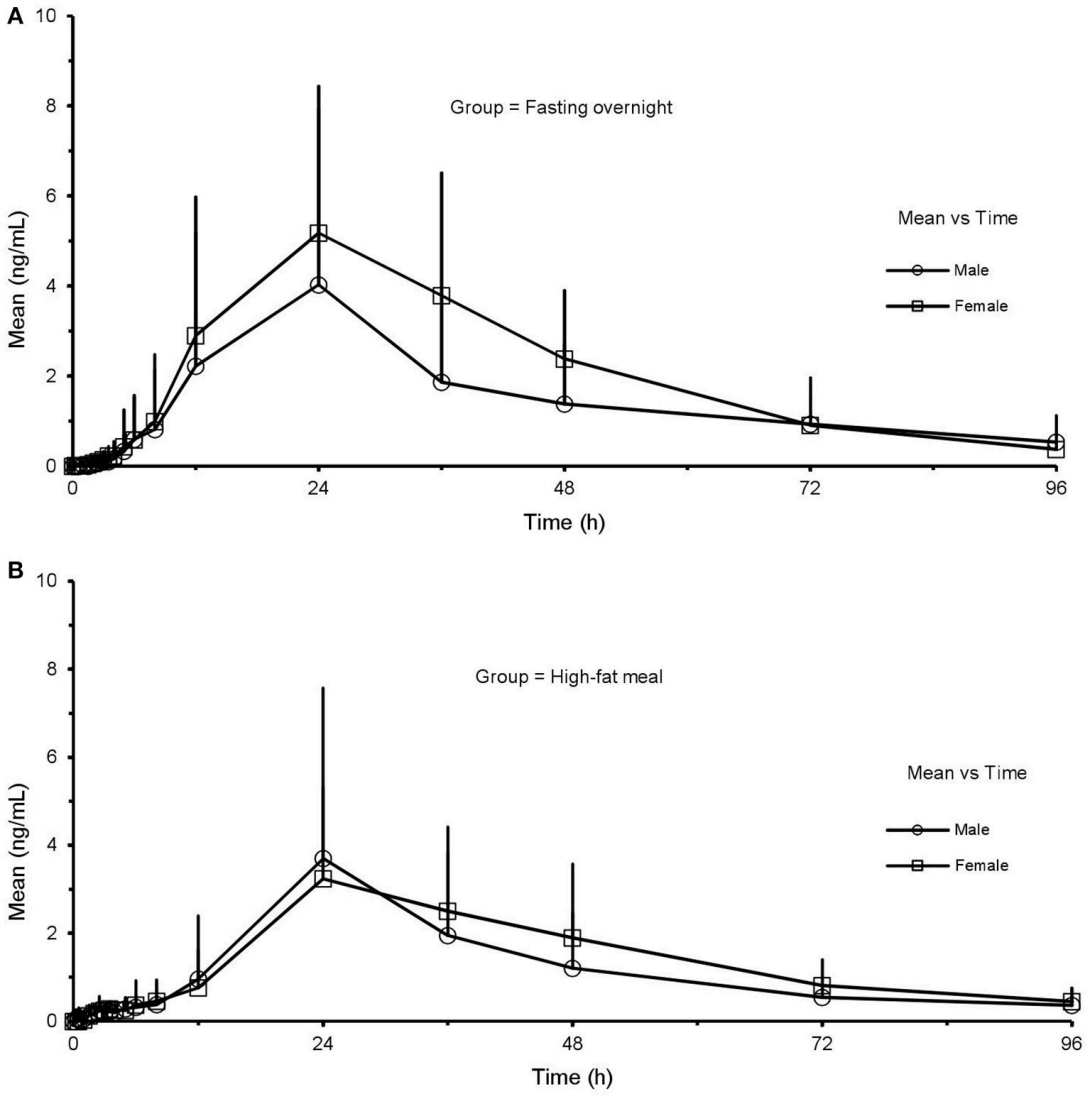
Mean (SD) plasma concentration-time profile of 20(S)-PPD after oral administration of CK 200 mg under fasting overnight **(A)** and after a high-fat meal **(B)** in male (*n* = 12) and female (*n* = 12) subjects, respectively. CK, compound K; PPD, protopanaxdiol; All values are presented as mean ± standard deviation.

It was observed 20(S)-PPD showed slight sex-related differences in both states (Figure 5). As shown in Table 5 and Table 6, there were significant sex-related differences on AUC_last_ either in the FO or HF group (119.6 ± 124.0 vs. 202.9 ± 88.8 h·ng·mL^−1^, *p* = 0.033; 27.0 ± 7.4 h vs. 36.3 ± 6.8 h, *p* = 0.290, respectively).

## Discussion

Data available on the PK profile of ginsenoside CK in humans are limited, and it should be noted that this study was included in a series of phase I clinical trials researching the pharmacokinetics of oral monomer ginsenoside CK, performed in healthy volunteers for the first time. This trial described the impacts of food and sex on the pharmacokinetics of ginsenoside CK and its metabolite 20(S)-PPD.

On one hand, the exposure parameters C_max_ and AUC of CK remarkably increased with the concurrent administration of a HF breakfast with CK Tablets 200 mg. The FDA guidelines state that a food effect is established when: “…90 percent CI for the ratio of population geometric means between fed and fasting treatments, based on log-transformed data, is not contained in the equivalence limits of 80 to 125 percent for either AUC_inf_ (AUC_last_ when appropriate) or C_max_” (FDA, 2002). These criteria, combined with our results, demonstrated that HF significantly increases the extent and rate of absorption of ginsenoside CK. On the other hand, the phenomenon of sex dimorphism in CK pharmacokinetics was observed in this trial. Although no statistically significant differences in the PK parameters between male and female subjects were observed in the FO state, the exposure of CK was higher in women than in men. This difference was particularly significant after a HF meal, with higher C_max_, AUC_last_, and AUC_inf_ of CK and lower V/F and Cl/F in female subjects than in male subjects, which were consistent with phenomena observed from our previous animal experiments and clinical trials (Supplementary Figure S1 and Supplementary Table S1). It is obvious that these sex-related differences could not be simply explained by the variance in body weight between men and women. Also, it is difficult to infer which process the food impact on *in vivo* through these parameters. In addition, variations of 20(S)-PPD induced by either food or sex were so slightly that it is difficult to determine the food/sex-related effects on it. This could possibly be because the exposure of PPD in the plasma was relatively low, with a large interindividual variation simultaneously.

Results from a two-way ANOVA test showed that there was no significant interaction on the PK of CK between the two independent factors, sex, and food. The interaction plot was particularly useful for determining interaction effects, as it displays means for the levels of one factor on the x-axis and a separate line for each level of another factor. According to the plots, there was a tendency of the two lines to intersection, for they were not completely parallel. This suggests that food and sex may have a potential interaction effect, but it had not reached to the statistical degree.

The exact mechanism responsible for increasing ginsenoside CK exposure in the presence of a HF meal is unknown and may be a result of increasing solubilization and dissolution in the intestinal fluids. The basal gastric secretion can increase up to 5-fold after a HF food (Welling, 1996), which may improve the dissolution rate and lead to increased solubility by increasing wetting of drugs and micellar solubilization. This speculation is consistent with previously reported results (Paek et al., 2006). Moreover, available *in vitro* and *in vivo* data suggest that CK undergoes P-glycoprotein (P-gp)-mediated intestinal efflux (Yang et al., 2012; Zhang et al., 2012; Li et al., 2014). For an orally administered drug, the first-pass is the brush-border membrane of intestinal enterocytes, before it enters the circulation. P-gp is expressed in the apical membrane of the entire intestine, from duodenum to rectum, thereby causing the reduced bioavailability of many substrate drugs (Canaparo et al., 2007; Zakeri-Milani and Valizadeh, 2014). Konishi et al. reported that the monoglyceride in the HF could inhibit the activity of P-gp (Konishi et al., 2004) and, thus, could alter the pharmacokinetics of substrate drugs. To date, the interactions between CK and other transporters that play an essential role in absorption, metabolism, distribution, and excretion have not been studied. Therefore, the intricate drug-transporters and food-transporters interactions may be partially attributable to explain the food-related impact on CK in this trial.

Sex dimorphism in pharmacokinetics is not a rare phenomenon. FDA retrospectively analyzed 300 drugs approved from 1995 to 2000, of which 11 drugs had sex-dependent differences >40% (Anderson, 2005). Over the past 20 years, some international communities and organizations have put forward a series of important regulations requiring clinical trials, pre-clinical animal studies, and even cell experiments to take sex into consideration (USA, Report to Congressional Requesters, 2000; NIH policy guideline on the inclusion of women minorities as subjects in clinical research, 2001; Bren, 2005; Clayton and Collins, 2014; Couzin-Frankel, 2014). This shows that researchers have gradually begun to recognize the importance of sex in drug development and clinical applications. However, the mechanisms behind the sex-related differences are yet to be fully understood. Physiological factors, such as, body weight, tissue size, glomerular filtration rate, intestinal motility, and protein levels, including transporters and metabolism enzymes, contribute to the sex-related differences observed when assessing drugs *in vivo* (Gandhi et al., 2004). Additionally, the levels and modes of secretion of hormones differ between males and females, therefore, hormones are also thought to play a role in the differences observed between sexes (Li et al., 2013). A recent study showed that gastric mucosal blood flow is higher in male than in female rats, and is reduced in male rats by estrogen administration (Shore et al., 2017), which lead us to consider whether hormones are involved in the regulation of the disposition of CK. Nevertheless, additional mechanisms could simultaneously be involved, with different effects on blood concentrations of the parent drug and its metabolites. The mechanism responsible for the sex-induced differences in CK undoubtedly needs further *in vivo* and *in vitro* studies to be determined. In a word, Ginsenoside CK Tablet is expected to be a precise drug for female RA patients, as it is known that the incidence of RA in women is two to three times higher than in men (Kurko et al., 2013).

Although this trial did enrich the PK research and provide novel directions for CK investigations, its limitations are also evident. First of all, this study was performed on a small sample of healthy subjects with an average age of 23 years, while the prevalence and incidence of RA increase with age. Thus, the PK characteristics obtained from this trial may not be fully applicable to populations of other age. Secondly, the single dosage form of CK at this stage restricts more comprehensive studies in human beings, which could thoroughly investigate the absorption, distribution, metabolism, and excretion. Since food or sex can affect the absorption parameters, and, simultaneously, the distribution/metabolism/excretion parameters, it is difficult to infer the impact on the organs accurately, without an estimate of bioavailability. Third one is, the test meal in the present trial may differ significantly from the daily diet. Although based on FDA (FDA, 2002), the effect of the HF meal represents the maximization of food effect, a true “food” effect would require more than one comparison between fasting and a high-fat meal. Moreover, this study can only provide some preliminary results, with a large number of experiments being required to verify the true mechanism behind the food and sex-related differences. As ginsenoside CK is a drug product for INDs applications, a series of *in vitro* and *in vivo* studies have been completed, and phase II clinical trials are currently due to begin. At the same time, several experiments are being conducted in our laboratory, which may contribute to explaining the food and sex-related differences in the pharmacokinetics of CK. In summary, it is worth noting that food and sex must be taken into consideration in future studies as two key points, which could help guide the clinical application of CK. The present study can also give some insights to scientific researchers in other ways.

## Conclusions

A single 200 mg dose of Ginsenoside CK Tablets appeared to be well tolerated, with no serious AEs being observed in this food-effect study. As two independent factors, both food and sex had an impact on the disposition of CK *in vivo*, but not a significant interaction effect. Food intake shortened the peak time of CK in the plasma and increased its exposure *in vivo*. Plasma levels of CK were higher in females compared to males. According to the present study, food and sex are two key factors that must be considered in follow-up studies, as they could potentially guide the clinical application of CK.

## Ethics statement

This study was carried out in accordance with the recommendations of the Declaration of Helsinki and International Conference of Harmonization guidelines for Good Clinical Practice, the ethics committee of the Third Xiangya Hospital affiliated to Central South University. All human participants gave written informed consent in accordance with the Declaration of Helsinki. The protocol was approved by the ethics committee of the Third Xiangya Hospital.

## Author contributions

LC and LZ were responsible for the study design, process of samples, data analysis, and article writing. YW assisted with the study design and data analysis. GY, JH, GZ, and JL were involved in the implementation of trials. ZT and YW participated in chromatographic analysis. DO was the principal investigator of this trial and was involved in the study design and data collection. All authors earnestly reviewed and approved the final version for this manuscript, and assume overall responsibility for the accuracy of data analysis and reporting.

### Conflict of interest statement

The authors declare that the research was conducted in the absence of any commercial or financial relationships that could be construed as a potential conflict of interest. The reviewer BND and handling Editor declared their shared affiliation.

## Abbreviations

G-CK: ginsenosides compound K
CK: compound K
PK: pharmacokinetic
FO: fasting overnight
HF: high-fat.
