# Supplementary material for: Food and Sex-Related Impacts on the Pharmacokinetics of a Single-Dose of Ginsenoside Compound K in Healthy Subjects
Source: Front Pharmacol. 2017 Sep 13;8:636. doi: 10.3389/fphar.2017.00636 (PMC5602130; doi:10.3389/fphar.2017.00636)
Supplement: Supplementary file 1 [file Table1.doc]

**Supplementary Table 1.** Sex differences of ginsenoside compound K in human (n = 12).

| **Parameter** | **Male** | **Female** |
| --- | --- | --- |
| C_max_ | 601.00 ± 182.74 | 872.56 ± 426.62* |
| t_max_ | 3.19 ± 1.25 | 2.58 ± 0.67 |
| t_1/2_ | 4.15 ± 1.90 | 5.66 ± 2.04* |
| AUC_last_ | 3604.22 ± 1322.99 | 4493.91 ± 2031.04* |
| AUC_inf_ | 3679.97 ± 1357.09 | 4666.06 ± 2143.26* |

* Compared with Male, *p* < 0.05
