# Supplementary material for: Food and Sex-Related Impacts on the Pharmacokinetics of a Single-Dose of Ginsenoside Compound K in Healthy Subjects
Source: Front Pharmacol. 2017 Sep 13;8:636. doi: 10.3389/fphar.2017.00636 (PMC5602130; doi:10.3389/fphar.2017.00636)
Supplement: Supplementary file 2 [file Image1.PDF]

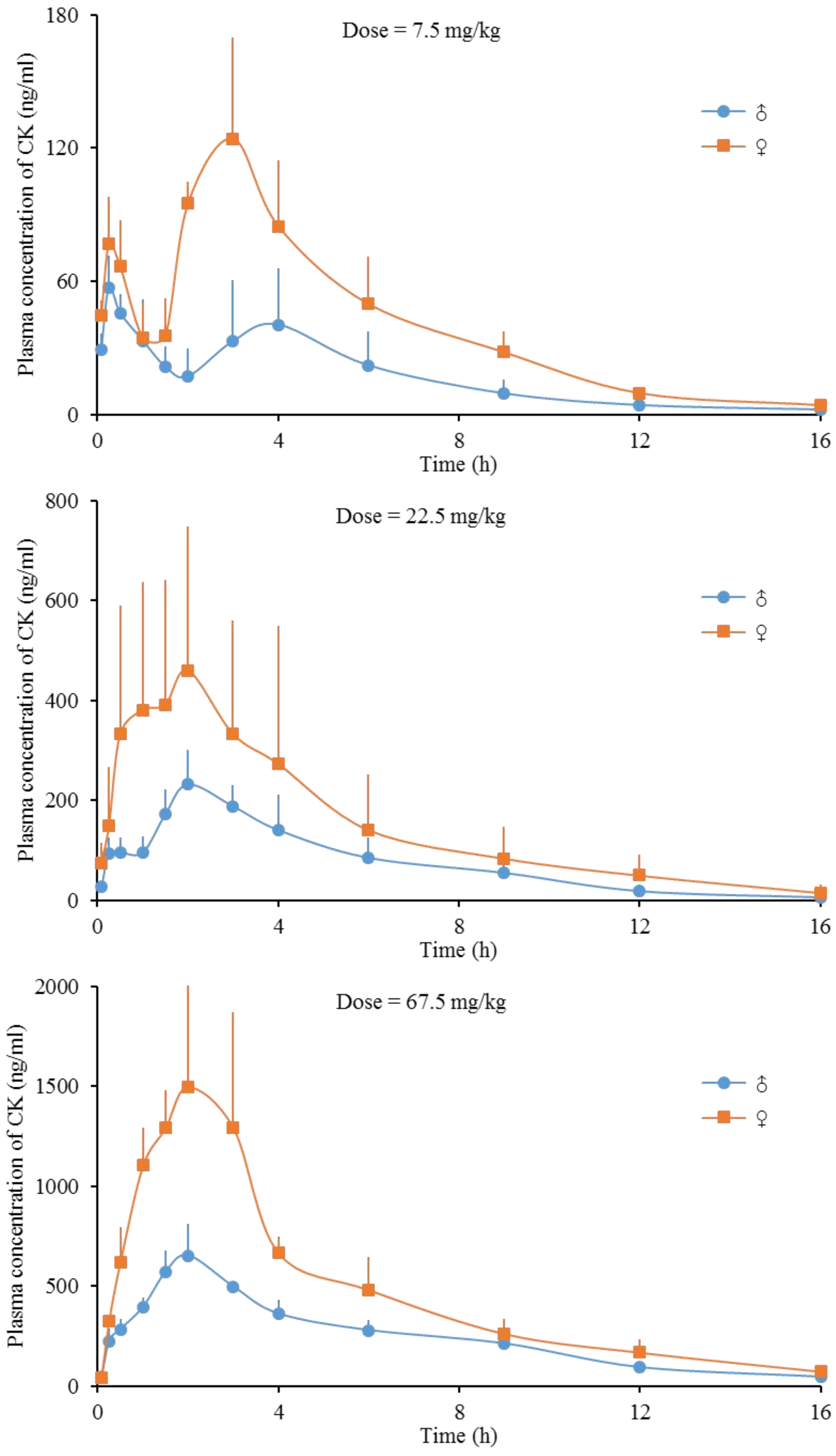

Supplementary Figure 1. Pharmacokinetic profiles (Mean ± SD) of CK in rats after intragastric administration of CK. SD, standard deviation; CK, compound K.
